# Preface

**DOI:** 10.1093/jac/dkac249

**Published:** 2022-09-06

**Authors:** Alan P Johnson, Roland Koerner, John D Perry

In response to the global threat to public health posed by antimicrobial resistance (AMR), the World Health Assembly formulated a Global Action Plan (GAP) in 2015. As part of this programme, countries were encouraged to develop National Action Plans to tackle AMR that are aligned with the GAP but adapted to national priorities and country-specific contexts. In this Supplement, information is provided on actions that have been initiated in a number of countries, particularly with regard to antibiotic prescribing, the development and use of local or international treatment guidelines and the availability of susceptibility data for two important respiratory pathogens, *Streptococcus pneumoniae* and *Haemophilus influenzae*. The articles highlight the varying approaches used and some of the practical challenges to establishing and undertaking relevant surveillance. It is hoped that sharing these experiences will be of value to clinicians, microbiologists, public health professionals and policymakers involved in similar activities in other parts of the world.

